# Great Tit ( *Parus major* ) Nestlings Have Longer Telomeres in Old‐Growth Forests Than in Young Forests

**DOI:** 10.1002/ece3.70823

**Published:** 2025-01-12

**Authors:** Ronalds Krams, Dina Cīrule, Maris Munkevics, Sergejs Popovs, Priit Jõers, Jorge Contreras Garduño, Indrikis A. Krams, Tatjana Krama

**Affiliations:** ^1^ Latvian Biomedical Research and Study Centre Riga Latvia; ^2^ Department of Biodiversity, Institute of Life Sciences and Technologies Daugavpils University Daugavpils Latvia; ^3^ Chair of Plant Health, Institute of Agricultural and Environmental Sciences Estonian University of Life Sciences Tartu Estonia; ^4^ Institute of Food Safety, Animal Health and Environment "BIOR" Riga Latvia; ^5^ Section of Ecology, Faculty of Medicine and Life Sciences University of Latvia Riga Latvia; ^6^ Statistics Unit, Faculty of Medicine Riga Stradins University Riga Latvia; ^7^ Institute of Molecular and Cell Biology University of Tartu Tartu Estonia; ^8^ Escuala Nacional de Estudios Superiores National Autonomous University of Mexico Morelia Mexico; ^9^ Institute of Ecology and Earth Sciences University of Tartu Tartu Estonia

**Keywords:** development, habitat quality, hematological stress, *Parus major*, telomeres

## Abstract

Modification and deterioration of old‐growth forests by industrial forestry have seriously threatened species diversity worldwide. The loss of natural habitats increases the concentration of circulating glucocorticoids and incurs chronic stress in animals, influencing the immune system, growth, survival, and lifespan of animals inhabiting such areas. In this study, we tested whether great tit ( *Parus major* ) nestlings grown in old‐growth unmanaged coniferous forests have longer telomeres than great tit nestlings developing in young managed coniferous forests. This study showed that the patches of young managed coniferous forests had lower larval biomass than old‐growth forests. Since insect larvae are the preferred food for great tit nestlings, the shortage of food may divert energy resources away from growth, which can show up as physiological stress, often raising the heterophil/lymphocyte (H/L) ratio. The H/L ratio revealed a significant difference in stress levels, being the highest in great tit nestlings developing in young‐managed pine forests. We also found that the development of great tit nestlings in young managed forests had significantly shorter telomeres than in old‐growth forests. Although nestling survival did not differ between the habitats, nestlings growing up in old‐growth forests had greater telomere lengths, which can positively affect their lifespan. Our results suggest that the forest habitats affected by industrial forestry may represent ecological traps, as the development of young birds in deteriorated environments can affect the age structure of populations.

## Graphical abstract

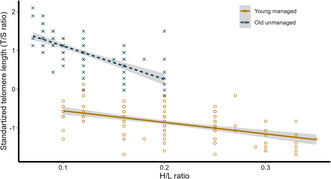

## 1 Introduction

When experiencing stress, organisms often cannot respond appropriately to additional stressors to counteract their consequences (Collier, Renquist, and Xiao 2017; Koolhaas et al. 1999; Steinberg 2012). Stress often causes alterations in homeostasis and intensifies energetic trade‐offs between competing organismal functions such as reproduction, immunity, development, and growth (Krams et al. 2011, 2017; Lancaster, Morrison, and Fitt 2017; Luoto 2019). Environmental factors can further increase differences in the availability of growth‐related resources, which may have long‐lasting consequences for physiological functions and fitness parameters later in life (Krams, Krams et al. 2020; Stearns 1992). Although organisms can efficiently regulate their internal state in response to acute stress (Zane, Ensminger, and Vázquez‐Medina 2021), stress induced by habitat quality incurs more extended consequences, causing long‐lasting chronic stress (Cīrule et al. 2017; Crawford et al. 2022; Krams et al. 2021; Krams, Mennerat et al. 2022; Krams, Krama et al. 2022).

Chronic stress usually raises energetic demands, increasing the overall demand for carbohydrate‐based fuel and shifting the metabolic balance away from anabolism that produces nitrogen‐rich (N) proteins necessary for growth (Hawlena and Schmitz 2010a, 2010b; Rinehart and Hawlena 2020). Under such circumstances, the body transforms proteins into carbohydrates (Hawlena and Schmitz 2010a), thus slowing down growth and development. Stress‐induced deficiency of proteins has long been known to impair immune function and increase the susceptibility of organisms to diseases (Li et al. 2007). Proteins and amino acids play important roles in activating lymphocytes, natural killer cells, and macrophages; regulating cellular redox state, gene expression, and lymphocyte proliferation; and producing antibodies and cytokines (Kelly and Pearce 2020). Thus, protein malnutrition decreases immune function, thereby decreasing life span (Kitada et al. 2019).

The heterophil/lymphocyte (H/L) ratio provides a reliable and widely used indicator of stress (Gross and Siegel 1983; Maxwell 1993; Aguirre et al. 1995; Ots et al. 1998; Davis et al. 2008). The H/L ratio is known to increase in response to various stressors, including starvation (Averbeck 1992; Vleck et al. 2000). Increasing evidence shows that variation of leukocyte counts can predict bird survival during the reproductive and non‐reproductive seasons (Hõrak et al. 1999; Lobato et al. 2005; Hylton et al. 2006; Kilgas et al. 2006). Survival is directly linked to the efficiency of the immune system. Immunity has been shown to depend on resources available during individual development (Krams et al. 2019; Luoto et al. 2021; Rubika et al. 2020). Longevity cannot be possible without a fully developed and properly working immune system to provide stress resistance (Karagiannis et al. 2023). Immunosenescence, or the aging of the immune system, is a consequence of the continuous attrition caused by chronic antigenic overload and the organism's subsequent inability to efficiently remove its pathogenic burden (Asghar, Bensch et al. 2015; Asghar, Hasselquist et al. 2015; Sansoni et al. 2008).

Telomere shortening is a hallmark of cellular aging and has been implicated in various age‐related diseases, including cancer and neurodegenerative disorders (Haussmann et al. 2003; Rossiello et al. 2022). Telomeres are repetitive DNA sequences located at the ends of chromosomes that serve as protective caps to maintain the stability of genetic material (Blackburn 1991; Daniali et al. 2013). Telomeres shorten with cell divisions, and their shortening is exacerbated by cellular and extracellular stressors, such as oxidative stress, high energy demand, and diseases (Casagrande and Hau 2019). Environmental stress can activate the hypothalamic–pituitary–adrenal (HPA) axis, increasing the production of stress hormones such as cortisol (corticosterone), while chronic exposure to stress hormones has been linked to telomere shortening (Casagrande et al. 2020; Lin and Epel 2022). Several studies show higher stress‐induced corticosterone levels and higher oxidative stress in urban bird populations compared with rural bird populations (Chatelain, Drobniak, and Szulkin 2020; Fokidis, Orchinik, and Deviche 2009; Ibáñez‐Álamo et al. 2018; Isaksson et al. 2009), likely due to increased exposure to all kinds of pollution, including light and noise pollution and malnutrition.

In birds, telomere shortening has been linked with worse survival (Monaghan 2008; Pepke et al. 2023; Tricola et al. 2018), stress (Boonekamp et al. 2014; Lemaître et al. 2021; Tablado et al. 2022), parasites (Tschirren et al. 2021), individual quality, and fitness (Angelier et al. 2019; Casagrande and Hau 2019), as well as habitat quality and geographic distribution of species (Kärkkäinen et al. 2022; Pepke et al. 2023), which makes telomeres a biological indicator of short‐ and long‐term costs of inhabiting a certain ecological niche (Angelier et al. 2018; Heidinger et al. 2012). A study (Salmón et al. 2016) showed that brief exposure of great tit *(Parus major* ) nestlings to an urban environment during early development shortens telomeres more significantly than development in rural habitats and that there is a potential direct relationship between the relative length of telomeres and the birds' lifespan.

Previous studies have shown that passerine birds living in old forests are often in a better physiological condition than their conspecifics dwelling in heavily managed forests (Betts et al. 2018, 2022; Krams et al. 2010, 2021; Suorsa et al. 2003). It has been suggested that higher stress levels in passerines inhabiting highly fragmented young managed forests are due to malnutrition and a higher risk of predation (Krams, Krams, and Cernihovics 2001; Zanette and Clinchy 2020). It has been shown that willow tits ( *Poecile montanus* ) and Siberian tits ( *P. cinctus* ) had lower survival, higher H/L ratio, higher baseline corticosterone, and a less significant increase in corticosterone in response to capture in young forests than individuals in old forests (Cīrule et al. 2017; Krams et al. 2010). These findings can potentially explain the dramatic decline of some parid populations (Fuller et al. 2007; Kumpula et al. 2023; Lewis et al. 2007). In this study, we compared the amount of food resources available to great tit nestlings, their H/L ratios, and early‐life telomere lengths (TL) in young managed (low‐quality territories) and old unmanaged (high‐quality territories) coniferous forests (Cīrule et al. 2017). Although we expected similar clutch sizes between young and old forests, we predicted higher H/L ratios, smaller fledgling numbers, and longer telomeres in great tit nestlings reared in old unmanaged forests than nestlings reared in young managed forests due to malnutrition and higher stress levels in the nest boxes located in the young pine forests.

## 2 Materials and Methods

### 2.1 Study Area and Birds

The study was conducted near the town of Krāslava in southeastern Latvia (55°87′ N, 27°23′ E) in May and June 2018 and 2019. The study area covers approximately 12 km^2^ of mainly coniferous forests of different ages, from open clear‐cut areas and bogs to closed forests, dominated by Scots pine ( *Pinus sylvestris* ) and Norwegian spruce ( *Picea abies* ) (Rytkönen and Krams 2003). The data were obtained from 19 nests of great tits in young 40–50‐year‐old managed pine plantations with no understory and from 18 nests of great tits located in unmanaged 110–160‐year‐old mixed forests dominated by Norwegian spruce (Brūmelis et al. 2020; Cīrule et al. 2017). Nest boxes were located at least 450 m from one another.

The start of egg laying, clutch size, and onset of incubation were determined by weekly visits to the nest boxes. When nestlings were 15 days old, we measured their body mass (±0.1 g) and tarsus length (±0.1 mm) (Table 1) and collected 100 μL of blood from the tarsal vein from all the nestlings in each nest box for molecular sexing and 50 μL to estimate the H/L ratio (Krams, Mennerat et al. 2022; Krams, Krama et al. 2022). Following the methodology by Salmón et al. (2016), we evaluated the general body condition of great tit nestlings using the scaled body mass index (SMI), based on individual body mass and tarsus length.

**TABLE 1:** Summary of tarsus length and body mass of 15‐day‐old great tit nestlings, and clutch sizes and nestling counts of nests in old‐growth unmanaged and young managed coniferous forests.

Forest	Year	Tarsus, mm (mean ± SD)	Body mass, g (mean ± SD)	Clutch size (mean ± SD)	Nestling count (mean ± SD)
Young	2018	16.63 ± 0.27	19.03 ± 0.45	11 ± 0.63	10.82 ± 0.6
	2019	16.57 ± 0.30	19.03 ± 0.45	10.86 ± 0.69	10.29 ± 0.49
Old	2018	17.25 ± 0.43	19.31 ± 0.26	10.8 ± 0.63	10.8 ± 0.63
	2019	17.27 ± 0.29	19.38 ± 0.30	11 ± 0.89	10.67 ± 0.52

### 2.2 Nestling Survival

Survival of nestlings was evaluated as the number and proportion of juveniles fledged from each nest. Great tits typically fledged their nests between 19 and 21 days post‐hatch in this study.

### 2.3 Food Resources

In 2019, we estimated the food resources available in each forest patch using the frassfall method (Rytkönen and Krams 2003). In brief, frass production by larvae was measured using plastic funnels (diameter 35 cm) with a paper coffee filter (size 4) attached to each funnel. The filter lets rainwater go through, but frass produced by herbivory larvae is retained inside the filter. We used ten funnels in each study patch (*n* = 20). The funnels were attached to trunks of randomly chosen pines, and the distance between the funnels was c. 60 m. As soon as the first nestlings in the patch reached the age of 10 days, the funnels were placed for five days. The filters with the frass were preserved in a freezer. The frass production was determined by counting the frass items in each filter, and the average diameter of the frass items was determined by measuring randomly sampled frass items in each filter with an ocular micrometer. We estimated larval biomass from frass dry mass using an allometric relationship between frass diameter and dry mass (Rytkönen and Orell 2001) and the equation by Tinbergen and Dietz (1994). As we could not discriminate between frass produced by larvae of moths and sawflies (Zandt 1994), this part of the research estimated the total food resources available in each forest patch.

### 2.4 Leucocyte Counts

We prepared blood smears for differential white blood cell counts using the standard two‐slide wedge procedure (Krams et al. 2013, Cirule et al. 2012; Houwen 2002). The samples were air‐dried in the field, fixed in methanol (approximately 2 min), and stained with Wright‐Giemsa Quick stain in the lab. Smears were examined to obtain counts of lymphocytes and heterophils per 100 leucocytes. Obtained leucocyte counts were used for the calculation of the relative proportion of heterophils to lymphocytes (H/L ratios). All microscope counts of leucocytes were performed by the same person (DC). Repeatability of heterophil and lymphocyte counts was 0.88 and 0.91 (*ps* < 0.0001).

### 2.5 Telomere Length Measurement

Blood samples (100 μL) were taken from the tarsal vein of great tits and were stored in a SET buffer (0.015 M NaCl, 0.05 M Tris, and 0.001 M EDTA; pH 8) at −86 °C (Krams et al. 2012; Krams, Mennerat et al. 2022; Krams, Krama et al. 2022). Telomeres were measured in the red blood cells (RBCs) using a qPCR amplification method, a well‐established and validated approach in bird research (Criscuolo et al. 2009). We used the Applied Biosystems MagMAX DNA Isolation Kit (Thermo Fisher Scientific, Waltham) to isolate the DNA, following the manufacturer's protocol. The concentration and purity of extracted DNA were measured using a Qubit 2.0 Fluorometer and Qubit dsDNA HS Assay Kit (Life Technologies, Carlsbad, CA, USA).

qPCR was done to amplify the telomere and glyceraldehyde‐3‐phosphate dehydrogenase (GAPDH) sequences by using the following primers: Telomere forward tel1b (5′‐CGGTTTGTTTGGGTTTGGGTTTGGGTTTGGGTTT‐GGGTT‐3′) and reverse tel2b (5′‐GGCTTGCCTTACCCTTACCCTTACCCTTACCCTTACCCT‐3′); Great tit‐specific glyceraldehyde‐3‐phosphate dehydrogenase (GAPDH) forward (5′‐TGTGATTTCAATGGTGACAGC‐3′) and reverse (5′‐AGCTTGACAAAATGGTCGTTC‐3′). TL was measured as the T/S ratio between the telomere repeat copy number (T) and control gene copy number (S), where GAPDH was used as the control single‐copy gene. This approach provides a relative measure of TL, providing reliable values for within‐species comparisons in the great tit (Salmón et al. 2016). TL was measured relative to a reference sample using a Rotor‐Gene Q (Qiagen, Hilden, Germany).

To perform qPCR, we generally followed the conditions by Salmón et al. (2016). In brief, qPCR reactions were done using 5 ng of DNA with sets of primers Tel1b/Tel2b at a concentration of 200 nM/200 nM and GAPDH‐F/GAPDH‐R at 100 nM/100 nM, in a final volume of 25 μL and containing 12.5 μL of Supermix (Platinium SYBR‐green q‐PCR SuperMix‐UDG, Invitrogen). Habitats and sexes were randomly sorted, and assays were performed in different plates. The conditions for the qPCR were telomeres 10 min at 95°C, followed by 27 cycles of 15 s at 95°C, 30 s at 58°C, and 30 s at 72°C; GAPDH for 10 min at 95°C, 15 min at 95°C, 30 s at 60°C, and 30 s at 72°C. In each plate (telomere and GAPDH), a serial dilution of a reference DNA was run in triplicate to produce a standard curve. Efficiencies of the reference curve were always within an acceptable range for both telomeres and GAPDH (Mean ± SD, telomeres: 99.3 ± 1.87, GAPDH: 100.98 ± 1.41). Relative TL was calculated as follows: ((1 + Telomere efficiency) ΔCt telomere (control—sample)/(1 + GAPDH efficiency) ΔCt GAPDH (control—sample)) (Pfaffl 2001; Salmón et al. 2016). All samples were run in triplicate, and two DNA samples were included in each plate as a “golden” sample to account for inter‐plate variation. Mean inter‐ and intra‐plate variation of the Ct values were 2.59% and 0.51% for the telomere reactions and 1.40% and 0.35% for the GAPDH reactions. The repeatability intraclass correlation coefficient of the assays was 0.82, CI = [0.74, 0.876].

### 2.6 Molecular Sexing

Molecular sexing of all great tit nestlings in both forest types was done using primers P2 and P8, following Griffiths et al. (1998). PCR products were separated by electrophoresis in agarose gel stained with ethidium bromide and visualized under UV light. In birds, females are the heterogametic sex, having 2 different sex chromosomes (ZW), whereas males are the homogametic sex (ZZ). Because of this difference between the W and Z fragments, females displayed two bands (W and Z copies), while males displayed one band (two copies of the Z fragment) (Dubiec and Zagalska‐Neubauer 2006).

### 2.7 Data Analyses

We used standardized TL values in the analysis by subtracting the mean value and dividing by the standard deviation (Verhulst 2020). We fitted a linear mixed effects model to assess how standardized TL was affected by fixed factors: forest type (young managed or old unmanaged), proportion of heterophils to lymphocytes, nestling sex, SMI, and year of the study, and two‐way interactions between the forest type and all other variables. We also fitted linear mixed‐effect models to assess how H/L ratios were affected by forest type, nestling sex, SMI, and year of the study, set as fixed factors, and two‐way interactions between the forest type and all the other variables. We set nest box identification number as random intercept factors in both models. We used Levene's test to assess the homogeneity of variances and histograms of model residuals and the Shapiro–Wilk test to check for normal distribution of residuals. After that we used stepwise reduction of non‐significant variables and interactions to arrive at the final model, which had the lowest AIC value. We report the full model and the final model in the results.

In addition, we checked differences in larvae biomass between forests with the Mann–Whitney U test, and we checked if the number of eggs and number of nestlings at day 15 after hatching depended on season and forest type using generalized linear models with Poisson distribution. We also fitted binomial logistic regressions to compare the survival of nestlings between forest types and years of study. All analyses were performed in R version 4.1.0 (R Core Team 2021). Mixed‐effect models were fitted using package lme4 (Bates et al. 2015). All tests were considered significant at *p* < 0.05.

## 3 Results

### 3.1 Food Resources

**FIGURE 1:**
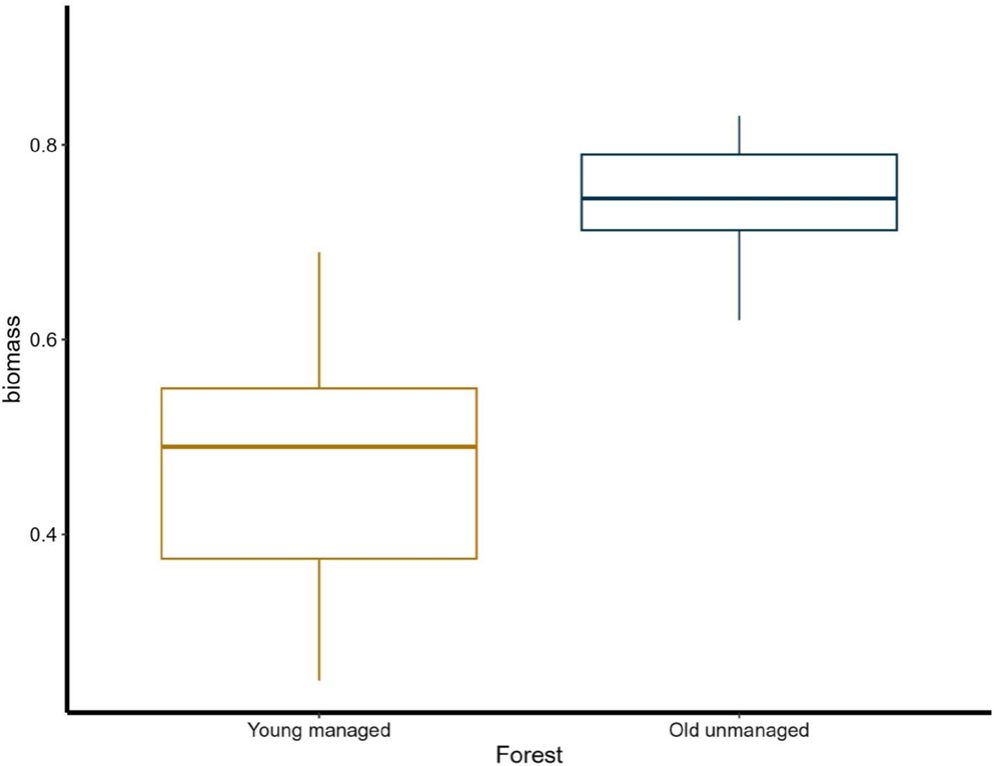
Boxplots showing larval biomass (g × m^−2^) in old‐growth unmanaged and young managed coniferous forests.

The amount of larval biomass was significantly (Mann–Whitney U test: W = 4, *p* < 0.001) higher in old unmanaged forest (biomass = 0.750 g × m^−2^; SD = 0.083, Figure 1) than in young managed forest (biomass = 0.472 g × m^−2^; SD = 0.148, Figure 1).

### 3.2 Hematological Stress

**FIGURE 2:**
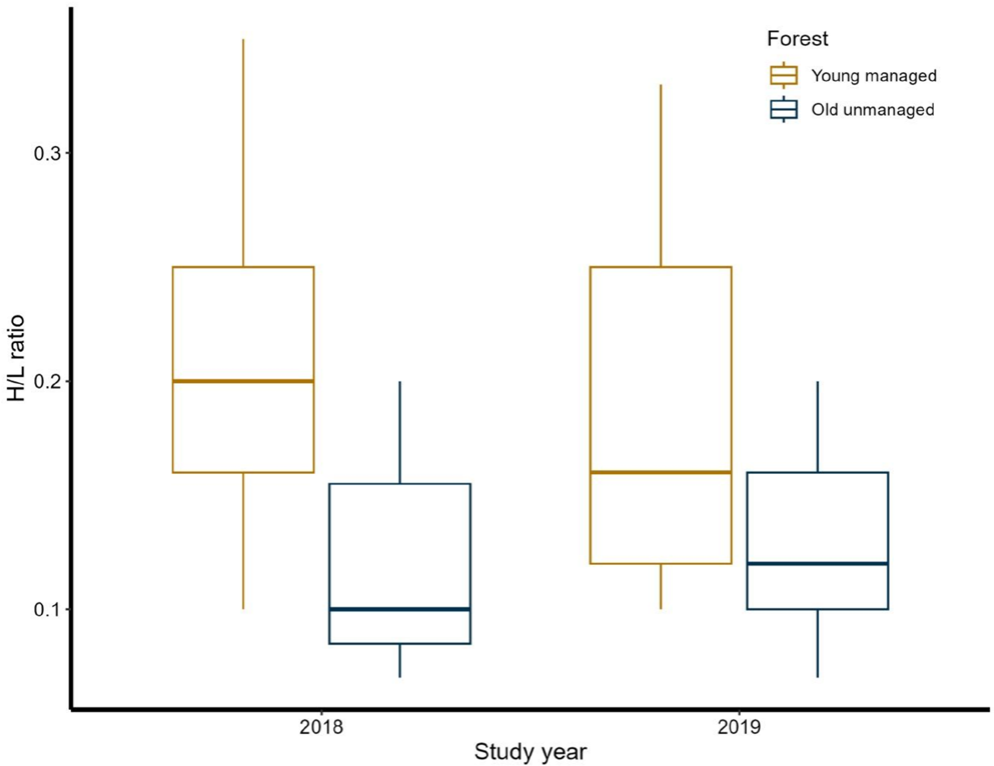
Boxplots showing the H/L ratios in 15‐day‐old great tit nestlings, grown in old‐growth unmanaged and young managed coniferous forests during two study years.

Nestlings from old unmanaged forests had a significantly (*p* < 0.001, Table 2, Figure 2) lower proportion of heterophils to lymphocytes (H/L ratio = 0.118; *SD* = 0.036, Figure 2) when compared to nestlings from young managed forests (H/L ratio = 0.202; *SD* = 0.066, Figure 2). Nestlings during the latter year (H/L ratio = 0.152; *SD* = 0.059, Figure 2) of the study also had significantly (*p* = 0.011, Table 2, Figure 2) lower H/L ratio compared to nestlings during the first year of the study (H/L ratio = 0.168; SD = 0.072, Figure 2). We also found significant interaction between the forest type and year of the study (*p* = 0.002, Table 2, Figure 2). Specifically, the effect of forest type on H/L ratio was more pronounced in the year 2019.

**TABLE 2:** Summary of the full and final model for H/L ratio in 15‐day‐old great tit nestlings grown in old‐growth unmanaged and young managed coniferous forests.

Model	Independent variables	Estimate (SE)	*t*	df	*p*
Full Model Response: H/L Ratio AIC = −706.11	Intercept	0.191 (0.150)			
	Forest (Old)	0.131 (0.248)	0.526	227.6	0.599
	Sex (male)	0.003 (0.009)	0.380	214.0	0.704
	SMI	0.001 (0.007)	0.158	226.1	0.875
	Season (2019)	‐ 0.039 (0.015)	−2.706	31.6	0.011
	Forest × Sex	−0.005 (0.013)	−0.385	215.6	0.701
	Forest × SMI	−0.012 (0.013)	−0.929	226.6	0.354
	Forest × Season	0.045 (0.021)	2.114	32.3	0.042
Final model Response: H/L Ratio AIC = −743.31	Intercept	−0.246 (0.097)			
	Forest (Old)	−0.100 (0.013)	−7.460	29.15	< 0.001
	Season (2019)	−3.073 (0.015)	−2.715	31.5	0.011
	Forest × Season>	−0.044 (0.021)	2.111	32.3	0.043
Random Factor	Nest ID	χ^2^ = 17.183			0.001

Abbreviations: df, degrees of freedom; SE, standard error.

The H/L ratio was not affected by body condition (*p* = 0.368), sex (*p* = 0.922), or any interactions between forest type and other factors (all *p*s > 0.29).

### 3.3 Telomere Lengths

**FIGURE 3:**
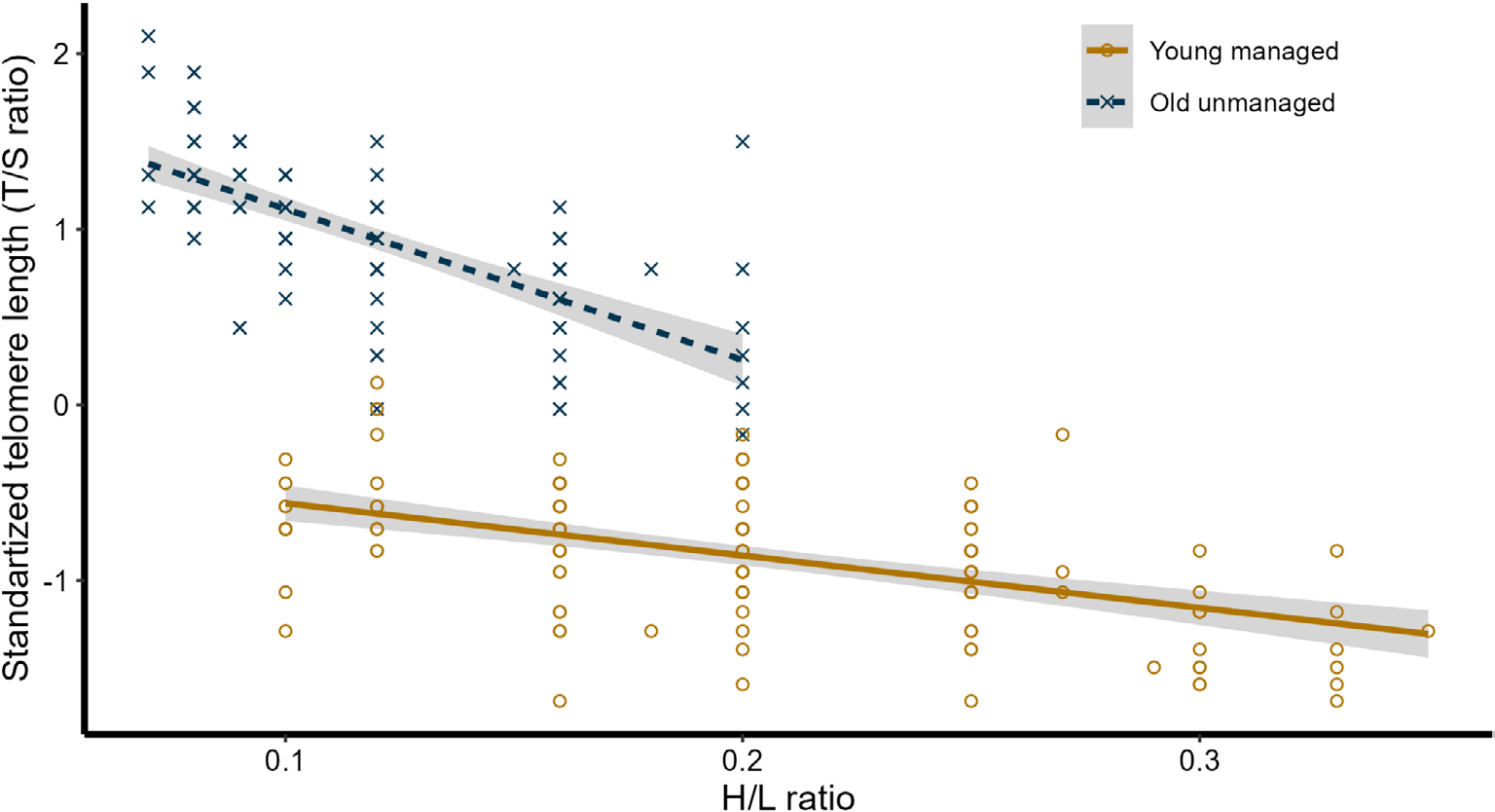
Relationship between standardized telomere lengths (T/S ratio, telomere length relative to a reference single‐copy gene) and H/L ratio in 15‐day‐old great tit nestlings, grown in old‐growth unmanaged (dark blue dashed line with × markers) and young managed (dark orange solid line with ○ markers) coniferous forests. Shaded areas represent 95% confidence intervals.

Nestlings reared in old unmanaged forests had significantly (*p* < 0.001, Table 3, Figure 3) longer telomeres (standardized T/S ratio = −0.865; *SD* = 0.372, Figure 3) compared to their conspecifics reared in the young managed forest (standardized T/S ratio = 0.960; *SD* = 0.448, Figure 3). TL was negatively related (*p* < 0.001, Table 3, Figure 3) to H/L ratio, and there was also a significant (*p* < 0.001, Table 3, Figure 3) interaction between the forest type and H/L ratio. TL was not affected by body condition (*p* = 0.721, Table 3), sex of the nestlings (*p* = 0.141, Table 3), year of the study (*p* = 0.448, Table 3), or any interactions of forest type with sex, body condition, and study season (all *P*s > 0.4, Table 3).

**TABLE 3:** Summary of the full and final model for standardized telomere length (T/S ratio, telomere length relative to a reference single‐copy gene) in 15‐day‐old great tit nestlings grown in old‐growth unmanaged and young managed coniferous forests.

Model	Independent variables	Estimate (SE)	*t*	df	*p*
Full Model Response: Stand. T/S AIC = 180.7	Intercept	0.104 (0.961)			
	Forest (Old)	3.467 (1.609)	2.155	234.2	0.032
	Sex (male)	0.059 (0.059)	1.477	216.6	0.141
	SMI	−0.017 (0.050)	−0.358	232.0	0.721
	H/L	−3.191 (0.460)	−6.937	227.4	< 0.001
	Season (2019)	−0.060 (0.078)	−0.767	35.4	0.448
	Forest × Sex	−0.041 (0.083)	−0.494	218.9	0.622
	Forest × SMI	−0.068 (0.083)	−0.812	233.5	0.417
	Forest × H/L	−5.430 (0.953)	−5.696	238.9	< 0.001
	Forest × Season	0.049 (0.112)	0.440	35.4	0.6626
Final model Response: Stand. T/S AIC = 152.4	Intercept	−0.246 (0.097)			
	Forest (Old)	2.205 (0.143)	15.459	204.6	< 0.001
	H/L	−3.073 (0.446)	−6.897	218.3	< 0.001
	Forest> × H/L	−5.411 (0.940)	−5.759	244.5	< 0.001
Random Factor	Nest ID	χ^2^ = 4.000			0.046

Abbreviations: df, degrees of freedom; SE, standard error.

### 3.4 Survival

The number of eggs did not differ significantly between forest types (Poisson regression: χ^2^ = 0.004, *p* < 0.951), or study years (χ^2^ = 0.0002, *p* < 0.988) (Table 1). There was also no significant difference between forests (Poisson regression: χ^2^ = 0.014, *p* < 0.905) and study years (χ^2^ = 0.090, *p* < 0.764) in the number of nestlings at day 15 after hatching (Table 1). We also found no differences between forest types (Binomial logistic regression: χ^2^ = 0.157, *p* < 0.692), and seasons (χ^2^ = 0.392, *p* < 0.531) in nestling survival rate.

## 4 Discussion

In this study, we compared the amount of food available, early‐life stress levels, and TL in altricial great tit nestlings during their post‐hatch development in young managed and old unmanaged coniferous forests. Clear‐cut forest areas, patches of young forests, increasing rates of forest fragmentation, and the removal of understory and other types of forest management and modification have decreased the quality of forest habitats and led to habitat loss in coniferous forests in Northern Europe. These changes have been linked to the population declines of many common forest species. Evidence suggests that habitat deterioration can increase stress‐induced corticosterone levels (Cīrule et al. 2017), which affects stress resistance (Krama et al. 2013; Krams et al. 2010), increases rates of immunosenescence (Bauch, Becker, and Verhulst 2013; Tablado et al. 2022; Vedder et al. 2022), decreases survival (Kumpula et al. 2023), and shortens TL of individuals in habitats of low quality (Angelier et al. 2015; Salmón et al. 2016).

The findings of this study indicate that post‐hatch development in a modified forest environment significantly increases the H/L ratio and leads to shorter telomeres in great tit nestlings. While the exact mechanisms by which poor environmental conditions affect TL remain unclear, our results suggest that growing up in a young, understory‐devoid forest may bring a negative impact on the lifespan of young great tits. TL is often regarded as a marker of cellular senescence (Stier et al. 2015; Vedder et al. 2022). However, it is unlikely that great tit nestlings would undergo accelerated aging during two weeks of post‐hatch development solely due to modified forest conditions. Instead, nestlings likely exhibit longer and more robust telomeres when raised in mature forests compared to modified forest patches because of habitat quality.

Previous studies have demonstrated that mature, unmanaged coniferous forests provide higher quality habitats than young, managed coniferous forest patches (Cīrule et al. 2017; Krama et al. 2013; Krams et al. 2010; Kumpula et al. 2023). Notably, social groups of parids experience lower within‐group competition and higher survival rates in mature, unmanaged forests, suggesting that young, managed forests may impose higher levels of stress and competition (Krams, Krama et al. 2022; Krams, Luoto et al. 2020).

Further research is needed to identify the factors that lead to the development of shorter telomeres during post‐hatch development in suboptimal environments, as well as to determine whether shorter telomeres directly impact survival and longevity. Prior studies present mixed findings: while Grunst et al. (2019) reported no significant relationship between TL and survival, Salmón et al. (2017) found that great tits with shorter telomeres were more likely to disappear from their habitats. Additionally, a meta‐analysis by Eastwood et al. (2023) found a general association between longer telomeres and enhanced survival in great tits. However, it should be tested whether birds with shorter telomeres might offset their post‐hatch stress and developmental challenges by dispersing to more productive and less stressful habitats later in life.

Most parid species do not require mature unmanaged forests as a prerequisite for reproduction (Cramp et al. 1993). However, it has been shown that clear‐cuttings and forest thinning measures in areas inhabited by willow tits increased natal and breeding dispersal distances and nearest neighbor distances (Kumpula et al. 2023). Habitat modifications and habitat loss have a significant role in the decline of willow tit populations. It has been found that forest management measures explain about 65% of the decrease in willow tit breeding density, which has made this once the 4th most common bird species in Finland endangered nowadays (Kumpula et al. 2023). Evidence suggests that the loss of old‐growth forests significantly decreases food available to parids both during the breeding and the non‐breeding season (Cīrule et al. 2017; Krams, Krams, and Cernihovics 2001; Kumpula et al. 2023). However, the direct reason for higher oxidative stress in parids inhabiting young managed forests remains to be shown because other factors can also be responsible for these parids' more significant telomere shortening. For example, adult birds may be more exposed to predation risk in young managed forests (Krams 2001, 1996; Krams et al. 2010). This can increase physiological stress, which may affect their offspring (Atema, van Noordwijk, and Verhulst 2022; Saulnier et al. 2023). The other reason for the observed difference in TL between the habitats may be a difference in the overall quality of individuals. Since telomere shortening is mainly controlled by genetic factors (Vedder et al. 2022), it could be tempting to assume that individuals breeding in young managed forests are less competitive and their genetic and phenotypic quality is lower than that in birds breeding in old‐growth forests. However, habitat quality has been shown to play a significant role in affecting TL (Salmón et al. 2016). Future research should focus on parent great tits breeding in old‐growth and young managed coniferous forests to disentangle whether the mechanism underlying telomere shortening is driven by genetic, hormonal or environmental factors (Hsu et al. 2023). The amount of food resources available to adults and nestlings should also be simultaneously controlled (Rytkönen and Krams 2003).

Although telomeres were longer in the old‐growth unmanaged forest, we found a significant and negative correlation between TL and H/L ratio, which was stronger in the old‐growth unmanaged forest than in the young managed forest. This result suggests that an increase in the H/L ratio, a marker of oxidative stress (Reichert and Stier 2017; Chatelain, Drobniak, and Szulkin 2020; Armstrong and Boonekamp 2023), has a significantly greater impact on TL in old unmanaged forests—a finding that may seem unexpected at first. It is widely accepted that ecosystem stability generally increases with species diversity, as supported by empirical data and theoretical studies (e.g., Lucini et al. 2020). However, as Sir Robert May highlighted (May 1972), diversity can sometimes have detrimental effects on ecosystem function. This phenomenon, known as the *diversity‐stability paradox*, has been observed when comparing high‐quality habitats, such as old‐growth deciduous forests, with lower‐quality habitats like managed coniferous woodlands (Mägi et al. 2009). Although great tits preferentially breed in old‐growth woodlands, reproductive success is often higher in lower‐quality habitats (e.g., Mägi et al. 2009). This counterintuitive outcome arises because the higher density of conspecific and heterospecific competitors in preferred habitats intensifies competition, leading to per capita food shortages and increased oxidative stress. As a result, even the most attractive habitats can act as ecological traps (Krams et al. 2021), where severe stress accelerates senescence and reduces reproductive success. Our findings suggest that shorter telomeres—commonly associated with stress and aging—can occur not only in less preferred habitats but also in high‐quality habitats. These results underscore the importance of further research in applied and conservation ecology to better understand the complex interplay between habitat quality, competition, and organismal stress.

The results of this study suggest that great tits breeding in young managed coniferous forests might be caught in ecological traps (Hale and Swearer 2016), when organisms mistakenly choose to live or breed in habitats that negatively affect their fitness. If this is the case, we would recommend installing nest boxes for great tits in old‐growth woods rather than in young managed forests (Krams et al. 2021; Krams, Mennerat et al. 2022; Krams, Krama et al. 2022), as growing up in the latter habitat may shorten telomeres of nestling great tits significantly more than posthatch development in old‐growth unmanaged forests. However, evolutionary change may also affect great tit populations in industrial forests so that birds can adapt to the human‐induced changes in their environments (Saulnier et al. 2022).

## 5 Conclusions

The great tit, one of the most common European birds, often chooses to nest in young forests, as long as nest boxes are available. Although the number of eggs laid and the number of nestlings indicate that this species does well in any type of forest, in this study we demonstrate that the amount of food available in young forests is lower than in old forests that have not been affected by forestry. This study reveals that great tit chicks experience more stress and have shorter telomeres when great tits nest in young forests affected by forestry activities. Our results show that the life span of nestlings of great tits raised in young forests could be significantly different from their lifespan if the birds had developed in old forests, which represent optimal conditions for the great tit chick development. However, a higher density of conspecific and heterospecific competitors in the most preferred old‐growth forests may accelerate cellular senescence, generating ecological traps.

## Author Contributions

**Ronalds Krams:** investigation (equal), methodology (equal), writing – review and editing (equal). **Dina Cīrule:** investigation (equal), writing – review and editing (equal). **Maris Munkevics:** formal analysis (equal), writing – review and editing (equal). **Sergejs Popovs:** investigation (equal), writing – review and editing (equal). **Priit Jõers:** formal analysis (equal), methodology (equal), writing – review and editing (equal). **Jorge Contreras Garduño:** conceptualization (equal), validation (equal), writing – review and editing (equal). **Indrikis A. Krams:** conceptualization (equal), formal analysis (equal), funding acquisition (equal), investigation (equal), methodology (equal), supervision (equal), writing – original draft (equal), writing – review and editing (equal). **Tatjana Krama:** conceptualization (equal), funding acquisition (equal), project administration (equal), supervision (equal), validation (equal), writing – original draft (equal).

## Ethics Statement

Ethical permission #87 was issued by the Food and Veterinary Service of the Republic of Latvia. Birds were caught and banded under bird banding permit #40 issued to Indrikis A. Krams by the Latvian Ringing Centre.

## Conflicts of Interest

The authors declare no conflicts of interest.

## Data Availability

The data that support the findings of this study are available from the Zenodo repository (https://doi.org/10.5281/zenodo.14062477).
